# Technical Note: A respiratory monitoring and processing system based on computer vision: prototype and proof of principle

**DOI:** 10.1120/jacmp.v17i5.6219

**Published:** 2016-09-08

**Authors:** Nicolas Leduc, Vincent Atallah, Patrick Escarmant, Vincent Vinh‐Hung

**Affiliations:** ^1^ Department of Radiation Oncology University Hospital of Martinique France

**Keywords:** external motion monitoring, optical tracking, left‐breast cancer

## Abstract

Monitoring and controlling respiratory motion is a challenge for the accuracy and safety of therapeutic irradiation of thoracic tumors. Various commercial systems based on the monitoring of internal or external surrogates have been developed but remain costly. In this article we describe and validate Madibreast, an in‐house‐made respiratory monitoring and processing device based on optical tracking of external markers. We designed an optical apparatus to ensure real‐time submillimetric image resolution at 4 m. Using OpenCv libraries, we optically tracked high‐contrast markers set on patients' breasts. Validation of spatial and time accuracy was performed on a mechanical phantom and on human breast. Madibreast was able to track motion of markers up to a 5 cm/s speed, at a frame rate of 30 fps, with submillimetric accuracy on mechanical phantom and human breasts. Latency was below 100 ms. Concomitant monitoring of three different locations on the breast showed discrepancies in axial motion up to 4 mm for deep‐breathing patterns. This low‐cost, computer‐vision system for real‐time motion monitoring of the irradiation of breast cancer patients showed submillimetric accuracy and acceptable latency. It allowed the authors to highlight differences in surface motion that may be correlated to tumor motion.

PACS number(s): 87.55.km

## I. INTRODUCTION

Respiratory motion has been a challenge in the accurate radiation therapy for moving tumors. In thoracic tumors, motion can be greater than 3 cm.^(1)^ Motion first results in a lack of accuracy at the time of CT planning, leading to errors in contouring. It also results in loss of precision during the phase of treatment, leading to undue irradiation of normal tissue and reducing the total dose received by the target. To address this latter problem, respiratory gating has been a method of choice, and several commercial systems have been made available over the past decade.

Internal gating, such as the RTRT system,^(2)^ uses implanted fiducial markers to precisely localize the tumor and its course during respiratory cycle, or to track the internal anatomy, such as the motion of bones through real‐time X‐ray portal imaging during irradiation.

External gating is based on the tracking of moving surrogate markers located on the patient's surface. The RPM system (Varian Medical Systems, Palo Alto, CA), the AlignRT (Vision RT Ltd, London, UK) or the novel Catalyst (C‐RAD AB, Uppsala, Sweden) system are representatives of those techniques. The RPM system is based on the monitoring of an infrared captor located on the patient's anterior abdominal surface. It allows amplitude or phase gating. The AlignRT^(3)^ or the Catalyst^(4)^ system use full, continuous surface imaging to account for motion, do not require markers, and may be associated to third‐party gating apparatus. Other use similar optoelectronic localization technology,^5^, ^6^, ^7^ or derive from spirometer techniques.^(8)^ Combining the two approaches, the Novalis system (Brainlab AG, Feldkirchen, Germany) uses both X‐ray imaging and external signals to assess tissue motion.

These systems mainly perform free‐breathing gating or breath‐hold gating. Limitations include: the choice of distant and limited surrogates that may not represent the real motion of tumor in the complex course of breathing thorax; the price and complexity of setup. Due to lack of point correspondence in dynamic surface acquisition, imaging techniques used by the Catalyst system have difficulties providing real‐time 3D motion of specific reference points.^(9)^ For this study, we considered monitoring breast motion. Breasts are deformable structures capable of motion independent from chest bones.^(10)^ As some authors previously noticed, it is reasonable to assume surface motion to be a suitable surrogate for tumor motion,^11^, ^12^, ^13^ and surface imaging to be an adequate monitoring method for respiratory motion gating. Novel, low‐cost optical methods have recently emerged to measure patient external motion^(14)^ and show promising results, but still require manually pinpointing the initial location of markers.

In this article, we present the phantom and volunteer validation of a very low‐cost surface monitoring device able to detect and track the motion of multiple simultaneous markers set directly on the breast during breast cancer irradiation, as a first step of a potential respiratory gating system. We also assessed the existence of potential differences in the motion of the different parts of the breast, which could lead to inaccuracies in breast motion handling.

## II. METHODS

Madibreast is an in‐house‐made optical tracking system. It consists of a physical optical system with at least two lens‐camera subsystems set up with a precise geometry. Each subsystem is driven by a specific piece of software based on computer‐vision algorithms. A graphical user interface displays real‐time motion and data.

### A. Physical setup

**Figure 1:**
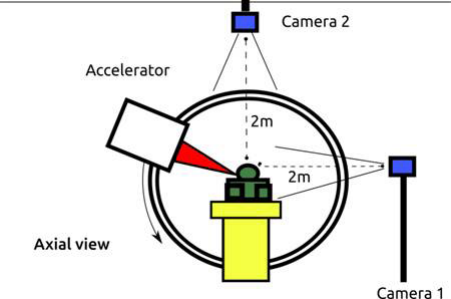
Physical setup of Madibreast in the treatment room.

The current physical set‐up is shown on Fig. 1. Camera 1 is set laterally of the patient and monitors anteroposterior (AP) and superior–inferior (SI) motion of the external part of the breast, which has been previously shown to move more than the other directions of motion.^(15)^ Camera 2 is set on the ceiling, monitoring lateral left–right (LR) motion and SI motion of the anterior surface of both breasts. All cameras are located at least 2–3 m from the patient to allow proper motion of the arm of the linear accelerator. Redundant information (SI direction) is computed separately. For validation purposes in this paper, we restricted the apparatus to the sole lateral camera. The markers tracked by Madibreast on the patients are high‐contrast centimetric markers made of paper or plastic material easily identified by the optical algorithm. Those markers can be set on the patient every day before irradiation or can last for the entire duration of treatment under waterproof conditioning. In the latter case, they can be used as positioning markers. Each camera is linked to a central multicore desktop computer.

### B. Optical apparatus

In this project, we performed image acquisition using low‐cost HD cameras with 1920×1080 px maximal resolution at 30 fps (Logitech C920 USB 2.0 webcam, Logitech International S.A., Newark, CA). This camera uses hardware H264 encoding to provide HD image at a rate up to 30 images per second. We dismantled the case and lens provided by the manufacturer to insert the Renoir CCD card into a custom aluminum case that allows attachment of C‐mount lenses. Various have been tested, from the mini 4× C‐mount Tamron lens (Tamron Europe GmbH, Cologne, Germany) up to Tamron AF70−300 mm f/4–5.6 zoom lenses. The aim was to capture a millimeter displacement at a 4 m distance with an accuracy of at least 2 pixels/millimeter.

### C. Optical tracking software

The core of Madibreast is based on the Open Source Computer Vision Library (OpenCV) with Python bindings. OpenCV is an open‐source, state‐of‐the‐art, practical set of software tools for computer vision that focuses on real‐time applications. It provides advanced functions for optical tracking of features under BSD license and is widely used for professional real‐time computer vision.^(16)^ We modified and included the initial algorithms into a Python program for our purposes. The algorithm can be summarized as follows. A shape detector first identifies the fiducial markers on the skin. The Shi‐Tomasi algorithm^(17)^ is then used for initial localization of points of interest on each marker. This corner detector analyzes areas of high contrast gradient and locates corners for optimal stability and accuracy of the process. Finally, those points are tracked in real time using the pyramidal Lucas‐Kanade method for optical flow computation.^(18)^ Code is written in Python language, with extensive use of multicore/multiprocess computation to achieve real‐time performance. Distortion filters and calibration ensure accurate rendering of distances by the camera.

### D. Graphical user interface

**Figure 2:**
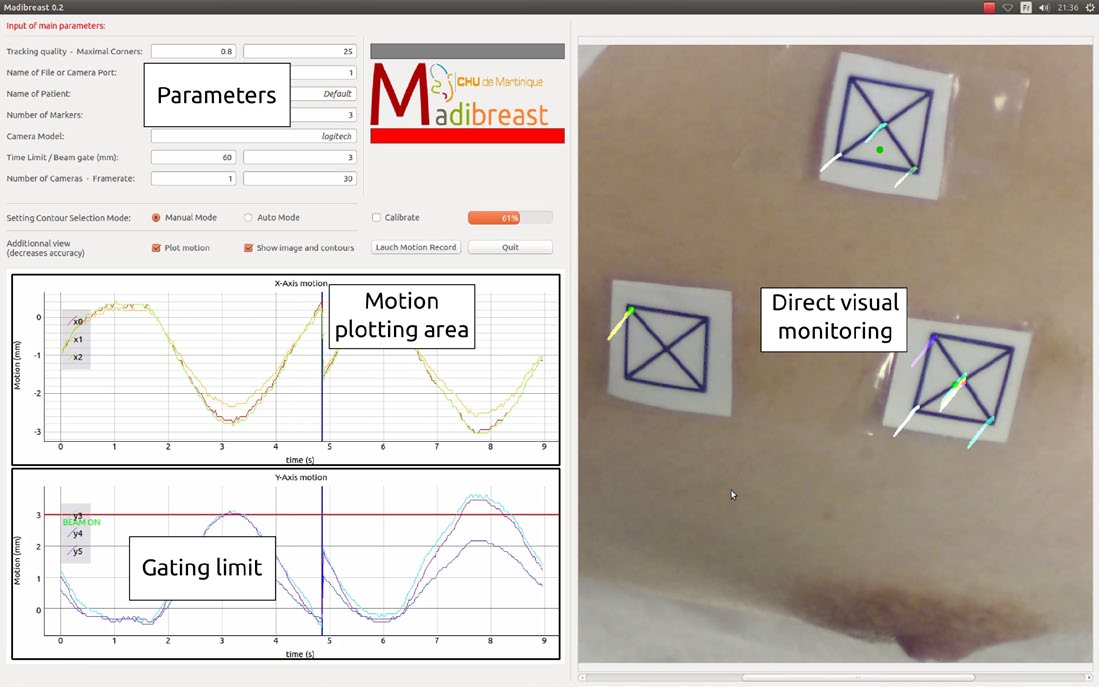
Graphical user interface of Madibreast. Various parameters can be selected to optimize monitoring. Plotting of motion as well as direct visualization are available (multimedia view).

In order to allow smooth and transparent use of the system by radiographers, we designed a very simple graphical interface (Fig. 2). The current position of fiducial markers, as well as the distance to the initial location, are displayed on the most recent picture acquired by the camera. Visual alerts when motion amplitude exceeds predefined values are available (gating).

### E. Validation system and protocol *E.1 Spatial accuracy*

It is well‐established that the accuracy in daily positioning is roughly of millimetric magnitude.^(19)^ As such, spatial accuracy of 1 millimeter should be sufficient for any gating device. We set our goal to this value.

#### E.2 Time accuracy

Monitoring of breathing motion may be particularly useful in clinical situations when keeping a steady respiratory pace is difficult (dyspnea or senility), as ample and fast motion of the chest is expected. We considered that it was reasonable to control breast motion up to a frequency f=30/min, with a maximum amplitude a=5 cm in any direction. As such, Madibreast was designed to track motion of maximal speed (2fa)=5 cm/s in a worst‐case situation. As speed increases, precisely detecting motion between two consecutive frames gets more challenging for Computer Vision methods.

#### E.3 Phantom validation protocol

**Figure 3:**
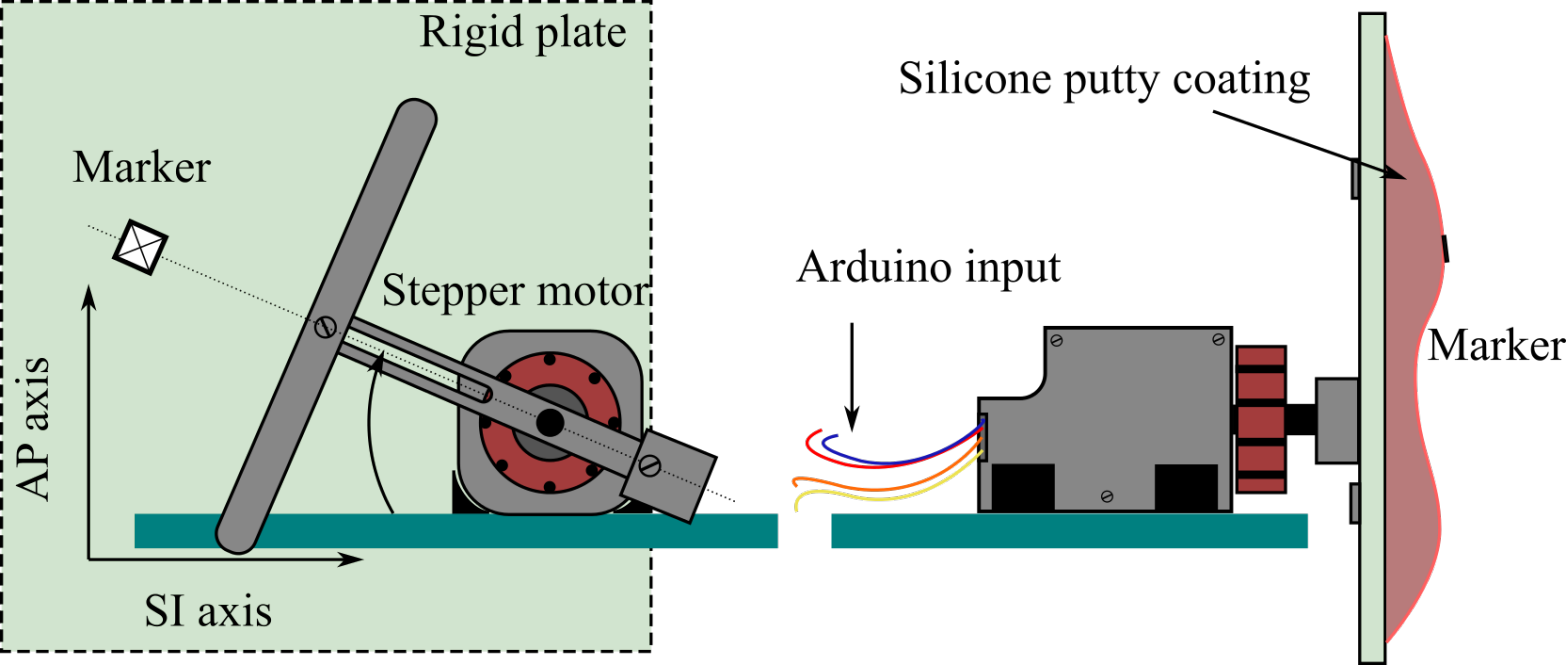
Front and side view of the in‐house‐made phantom.

To check the spatial and time accuracy of Madibreast, we designed an in‐house made mechanical phantom of thorax, able to simulate a various range of surrogates for breast motion. It consists of an Arduino UNO controller card driving a multiplied stepper motor (64×64 0.087°/step motor) that displaces the mobile parts of the phantom (Fig. 3). Feedback on motor position is provided by an optical encoder. Displacement of the attached fiducial marker is accurately driven in time and space by the user or by an algorithm. Motion of the patient's skin is modeled as a rotational motion of 90° from horizontal plane, followed by short respiratory pauses. As such, anteroposterior motion is a modified sine function of time. Expected mechanical accuracy is micrometric. Initial synchronization with Madibreast computation is performed by a start signal sent as serial command from the computer running Madibreast to the Arduino card (latency <10 ms).

We assessed robustness of the algorithm on a various range of breathing frequency and framerates. We defined failure as excessive long‐term drift (< 1 mm) or a discrepancy between the computations of two successive images (computed location minus actual location >0.5 mm).

#### E.4 Validation protocol on human volunteer

The final accuracy validation is an on‐site experiment on a volunteer in the irradiation room. One surface marker (point A) is set on the left breast. Breast motion is recorded during 50 s. The volunteer is instructed to change the amplitude and speed of her breathing pattern every 10 s. Time accuracy is checked comparing the value of system‐wide CPU clock right before image acquisition and after calculus of the tracked point. As the recording of pictures of motion is directly available through Madibreast, it is possible to check the spatial accuracy on the pictures without an external validation tool such as RPM. As such, we first visually identified the location of point A on each image for 40 s without looking at the results of tracking (single‐blind). Then, we compared this accurate result to the location as computed by the algorithm, using the least square method.

Finally, we set three different markers (A, B, and C) on one breast to assess possible differences in breast motion depending on the location of the marker. These were set respectively on the external‐caudal part of the breast, the median part, and close to the axillary area. We measured AP and SI motion of each location.

## III. RESULTS

A video file with a live, explanatory demo of Madibreast is available at https://vimeo.com/145533065.

### A. Validation on phantom experiment

#### A.1 Timing accuracy

We were able to acquire a new image, then complete tracking computations before acquiring the next image, in a mean 0. 0332 s (standard deviation (SD): 3.6 ms, max: 36 ms) with a HD 1920×1080 px resolution, during 5000 s, with one to three markers. System latency from image capture by the camera to the beginning of computation does not exceed 30 ms. Therefore, maximal delay between actual motion and then end of computation was always below 66 ms. A slight lag (mean 100 ms) was however noted between computation and display, probably due to the incomplete multi‐threading architecture of the program and the intrinsic limitation of Python language.

#### A.2 Spatial accuracy

**Figure 4:**
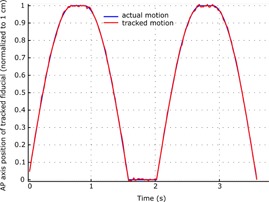
Normalized mechanical anteroposterior axis motion and motion as computed by the optical algorithm. Breathing pattern: 30 bpm, 330 ms respiratory pauses.

Tested with a Tamron AF70–300 mm f/4–5.6 lens with minimal zoom setting at a distance of 4 m, the captured area was 20×10 cm, with 10 pixels/mm resolution. Average spatial discrepancy between actual point of interest and tracking point was 1.5 px (0.15 mm) with a maximal 5 px (0.5 mm) during the same time span. Trajectories were fitted to the modified sine curves with R^2^ between 0.992 and 0.999 and root mean square error remained between 0.04 and 0.09 mm. Figure 4 plots an example of the median anteroposterior position of the fiducial and its estimated location computed with Madibreast, as a function of time.

Repetition of this pattern with various amplitudes and speeds showed no decrease of performance up to 5 cm/s and respiratory rate of 25 bpm.

### B. Validation on a human volunteer

**Figure 5:**
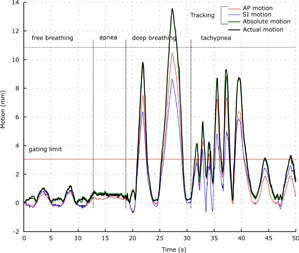
Validation on a human volunteer: example of computed and actual motion of the breast under different breathing patterns. As expected, actual motion and the tracked absolute motion are almost superimposable at this scale.

Markers were all correctly identified by Madibreast on the skin for good lightning conditions, with no use of infrared filters. There was no decrease of framerate when increasing the number of markers. 40 s (1,200 frames) of normal and deep breathing patterns have been recorded and manually analyzed. Mean difference between actual motion and computed motion was −0.01 mm (min −0.4 mm, max 0.5 mm, SD: 0.09 mm) in the SI axis, −0.01 mm (min −0.3 mm, max 0.5 mm, SD: 0.07 mm) in the AP axis and 0.01 mm (min −0.2 mm, max 0.7 mm, SD: 0.09 mm) in the LR axis. Tracking errors in the LK method occurred for higher breathing frequency (< 25 bpm) due to the increase in motion between two successive images. As can be inferred from Fig. 5, apnea, tachypnea, and deep‐breathing conditions have been captured adequately with submillimetric accuracy.

**Figure 6:**
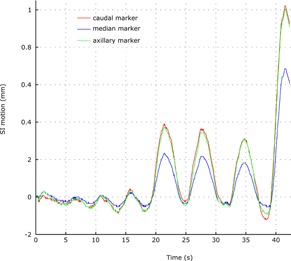
Superior–inferior axis motion of 3 markers set at three different locations on the left breast.

Results of a 3‐markers trial on the same volunteer are shown on Fig. 6. The superior–inferior motion shows discrepancy as high as 4 mm in the case of deep‐breathing pattern.

## IV. DISCUSSION

Experimental validation on phantom and human showed submillimeter accuracy with an acceptable latency in the computation and display of monitored points of interests with one camera. We were able to follow any location on the breast with a wide range of amplitude and respiratory frequency, including pathological patterns.

Multiple markers can be set on the skin to assess motion differences according to the initial location of tumor. On our volunteer's skin, noticeable differences in anteroposterior and superior‐inferior motion have been measured. Although motion difference of less than 1 mm occurred for normal breathing pattern, a difference up to 4 mm was found for dyspnea patterns. The ability of Madibreast to record signal from multiple markers on different breast locations (Fig. 5) may increase the correlation between processed external signal and actual tumor location,^(20)^ a possibility that does not exist with systems relying on one signal marker. Different results could be anticipated depending on cup‐size, ptosis, age, and the type of surgery.

Madibreast detects and tracks accurately external motion on the breast using low‐cost material and accessible open‐source, high‐level computer vision libraries. It allows immediate monitoring by visually displaying an immediate trace, which can alert that substantial motion could have occurred.

Limitations on this apparatus are still numerous. Failure of the algorithm occurs when lighting is insufficient, as well as for very rapid breathing patterns. Besides, latency increases with CPU occupation, and sometimes provides an occasional lag. This issue will be dealt with translation of the Python code into C/C++ code. It is also worth noticing that we used a very high resolution (1920×1080, i.e., 10px/mm), which is not necessary considering the requirements in spatial accuracy we set. The optical zoom capacities of the lens we used are sufficient to decrease the camera resolution to 1024×768 or less, and therefore improve speed of image transfer and computation.

## COPYRIGHT

This work is licensed under a Creative Commons Attribution 3.0 Unported License.
